# Drivers of desire for more children among childbearing women in sub-Saharan Africa: implications for fertility control

**DOI:** 10.1186/s12884-020-03470-1

**Published:** 2020-12-14

**Authors:** Bright Opoku Ahinkorah, Abdul-Aziz Seidu, Ebenezer Kwesi Armah-Ansah, Eugene Budu, Edward Kwabena Ameyaw, Ebenezer Agbaglo, Sanni Yaya

**Affiliations:** 1 School of Public Health, Faculty of Health, University of Technology Sydney, NSW Sydney, Australia; 2 Department of Population and Health, College of Humanities and Legal Studies, University of Cape Coast, Cape Coast, Ghana; 3 College of Public Health, Medical and Veterinary Sciences, James Cook University, Townsville, Queensland Australia; 4 Department of English, University of Cape Coast, Cape Coast, Ghana; 5 School of International Development and Global Studies, University of Ottawa, Ottawa, Canada; 6 The George Institute for Global Health, The University of Oxford, Oxford, United Kingdom

**Keywords:** Desire, Children, Reproductive health, Women, Public health, sub-Saharan Africa

## Abstract

**Background:**

Despite the extensive research on fertility desires among women the world over, there is a relative dearth of literature on the desire for more children in sub-Saharan Africa (SSA). This study, therefore, examined the desire for more children and its predictors among childbearing women in SSA.

**Methods:**

We pooled data from 32 sub-Saharan African countries’ Demographic and Health Surveys. A total of 232,784 married and cohabiting women with birth history, who had complete information on desire for more children made up the sample for the study. The outcome variable for the study was desire for more children. Multilevel logistic regression analysis was conducted. Results were presented using adjusted odds ratios (aOR), with their corresponding 95% confidence intervals (CI).

**Results:**

The overall prevalence of the desire for more children was 64.95%, ranging from 34.9% in South Africa to 89.43% in Niger. Results of the individual level predictors showed that women aged 45–49 [AOR = 0.04, CI = 0.03–0.05], those with higher education [AOR = 0.80, CI = 0.74–0.87], those whose partners had higher education [AOR = 0.88; CI = 0.83–0.94], women with four or more births [AOR = 0.10, CI = 0.09–0.11], those who were using contraceptives [AOR = 0.68, CI = 0.66–0.70] and those who had four or more living children [AOR = 0.09 CI = 0.07–0.12] were less likely to desire for more children. On the other hand, the odds of desire for more children was high among women who considered six or more children as the ideal number of children [AOR = 16.74, CI = 16.06–17.45] and women who did not take decisions alone [AOR = 1.58, CI = 1.51–1.65]. With the contextual factors, the odds of desire for more children was high among women who lived in rural areas compared to urban areas [AOR = 1.07, CI = 1.04–1.13].

**Conclusions:**

This study found relatively high prevalence of women desiring more children. The factors associated with desire for more children are age, educational level, partners’ education, parity, current contraceptive use, ideal number of children, decision-making capacity, number of living children and place of residence. Specific public health interventions on fertility control and those aiming to design and/or strengthen existing fertility programs in SSA ought to critically consider these factors.

## Background

It is estimated that, out of the 78 million added to the world population annually, about 97% originate from low- and middle-income countries [1]. Couples are having fewer children in recent times, and this may have led to a decrease in population growth in most high-income countries [2]. However, demographers seem to have concerns about the pattern of demographic development in low- and middle-income countries, due to the fact that, on the average Africans give 34 births per 1000 population, with a total fertility rate of 4.5 [3]. Countries in sub-Saharan African (SSA) account for more than 50% of global fertility [4]. Evidently, the Population Reference Bureau [3] estimates the fertility rate of SSA at 4.8, which is twice the global rate of 2.4.

Couples are able to achieve their fertility desires and pregnancy spacing by using modern contraceptives [5]. However, contraceptive use and other family planning strategies are considered to be low in SSA, and this has increased mistimed and unwanted pregnancies, as well as high youth dependency [6–8]. This reveals that desire for more children is of great concern for women in their reproductive ages in SSA [2]. It is estimated that, in SSA, contraceptive use among women in union is below 22%, compared with 86% in East Asia and 72% in Latin America and the Caribbean [9]. It has also been revealed that, in parts of SSA, more than 50% of women with four or more children still desire to have more children [10].

Deeply rooted in strong societal and personal factors [2, 11–14], desire for children is pivotal to family formation process in several communities of the world. Desire for more children is greatly driven by preference for large families, desire for sons, and the union’s stability [15–17]. A Demographic and Health Survey (DHS) based study observed that women in their reproductive ages in about sixty countries desired more children, particularly in Western and Middle Africa between 1998 and 2008; however, these women had an average of six children [10].

Theoretically, this work is situated within the demand-supply framework on fertility by Easterlin [18]. The framework has been applied to study fertility desire and intentions in various parts of the world. It posits that fertility desire(s) are influenced by both demand and supply factors. In the model, ‘Demand’ refers to the family size and composition a couple would have under ideal circumstances [18]. Its dimensions include the number of children, sex, and desirable spacing of surviving children, and these are influenced by the couple's personal preferences, the influence from community as well as the socio-cultural norms [18–20]. Supply, on the other hand, refers to the number of children the couples are able to bring forth. Based on these, a couple or woman will desire more children if they/she feel(s) that children are valuable and will serve as assets to them in the society in which they find themselves and vice-versa [18]. Those who do not desire more children may adopt various mechanisms to reduce childbirth, including contraceptive usage [21].

Bongaarts [22] provides an alternative implementation of the demand-supply framework for determining fertility proposed by Easterlin. The objective of Bongaarts framework was to simplify its application by changing some key features, while maintaining the original conceptual structure largely intact. Bongaarts proposed that fertility is a function of three determinants (supply of births, demand for births, and degree of preference implementation). Supply of births is measured as natural total fertility, which is the rate of childbearing that would prevail in the absence of deliberate efforts by couples to limit family size. Demand for births is measured as wanted total fertility, which refers to the rate of childbearing that would be achieved if all women were able to eliminate unwanted births. Degree of preference implementation is the net result of a decision-making process in which couples weigh the cost of fertility regulation and the cost of unwanted childbearing [22].

Majority of studies on fertility desire over the world have adopted a country-specific focus, paying attention to Nigeria [23], Iran [24], Nepal [25], and Uganda [2], with a few focusing on broader geographical areas such as East Africa [26], and Nigeria and Ghana [12]. Despite this extensive research, there is a relative paucity of literature on the fertility desires in SSA, as most countries in SSA are yet to feature in studies of this kind. The present study attempts to fill this gap by assessing the prevalence of desire for more children and its determinants among childbearing women in 32 countries in SSA.

## Methods

### Study design

We pooled data from the DHS of 32 sub-Saharan African countries. Specifically, we used data from the women’s file of the various countries. The DHS focuses on essential maternal and child health markers, including fertility preference [27]. The DHS employs a two-stage stratified sampling technique, which makes the survey data nationally representative [28]. A total of 232,784 married and cohabiting women with birth history who had complete information on desire for more children made up the sample for the study. Details of the methodology adopted by the DHS have been reported elsewhere [28]. Table 1 gives a detailed description of the study sample.

**Table 1:** Detailed description of the study sample

Survey Country	Survey Year	Sample (N)	Sample (%)
Angola	2015-16	5645	2.43
Benin	2017-18	8838	3.18
Burkina Faso	2010	11,842	5.09
Burundi	2016-17	9116	3.92
Cameroon	2018	6291	2.70
Chad	2014-15	10,055	4.32
Comoros	2012	2152	0.92
Congo	2011-12	6137	2.64
Congo DR	2013-14	11,479	4.93
Côte d’Ivoire	2011-12	5145	2.21
Ethiopia	2016	8598	3.69
Gabon	2012	3123	1.34
Gambia	2013	5561	2.39
Ghana	2014	4238	1.82
Guinea	2018	5905	2.39
Kenya	2014	7556	3.25
Lesotho	2014	3148	1.35
Liberia	2013	4709	2.02
Malawi	2015-16	12,353	5.31
Mali	2018	6913	2.97
Namibia	2013	2329	1.00
Niger	2012	8416	3.62
Nigeria	2018	24,342	10.46
Rwanda	2014-15	6405	2.75
Senegal	2010-11	8020	3.45
Sierra Leone	2013	8802	3.78
South Africa	2016	2310	0.99
Tanzania	2015-16	6886	2.96
Togo	2013-14	5167	2.22
Uganda	2016	9399	4.04
Zambia	2018	6512	2.80
Zimbabwe	2015	5392	2.32
**Total**	**-**	**232,784**	**100**

### Outcome variable

Desire for more children was the outcome variable. This was derived from the question “Would you like to have a (another) child with your husband/partner, or would you prefer not to have any more children with him?” It had five responses: “want a (another) child,” “want no more,” “cannot get pregnant,” “undecided,” and “don’t know.” Our outcome variable was computed from two of these responses, namely “want a (another) child,” coded as 1 and “want no more,” coded as 0. Hence, women who responded that they want another child were considered as having a desire for more children while those who responded that they want no more were considered as not having a desire for more children. Women who provided any other response (“cannot get pregnant,” “undecided,” and “don’t know”) were excluded because their responses were unclear about their fertility preference.

### Independent variables

The study used eleven independent variables, grouped into individual level and contextual level factors. The individual level factors included age, highest educational level, partner’s highest educational level, parity, current use of contraceptives, exposure to media (radio, television and newspaper/magazine), ideal number of children, decision making autonomy (decision on healthcare, decision on large household purchase and decision on visits to family or relatives), and number of living children. The contextual level factors were place of residence and wealth status. These variables were considered because of their statistically significant relationships with desire for more children in previous studies [2, 29, 30]. Details of how each of these variables were coded can be found in Table 2. Based on the findings of previous studies [2, 12, 21–26], we hypothesized that older women would be less likely to desire for more children compared to younger women; women with higher levels of education would be less likely to desire for more children compared to those with no formal education; women whose partners have higher levels of education would have lower odds of desiring for more children compared to those whose partners have no formal education. Other hypotheses that guided the analysis and results of the study were that the odds of desire for more children would decrease with increasing parity, wealth quintile, higher number of living children, contraceptive use and exposure to media. Women who consider 6 + as the ideal number of children, those who do not take decisions alone, and those who live in rural areas would be more likely to desire for more children.

**Table 2:** Desire for more children by explanatory variables (*n* = 232,784 weighted)

Determinants	Frequency (n)	Percentage (%)	Desire for more children %[95% CI]	Bivariate logistic regression		
				**cOR**	**Lower**	**Upper**
**Age**						
15–19	10,952	4.7	95.3[94.8–95.9]	1	-	-
20–24	39,321	16.9	91.3[91.0–91.8]	0.58^***^	0.52	0.63
25–29	52,378	22.5	83.2[82.7–83.7]	0.27^***^	0.25	0.29
30–34	46,206	19.8	67.6[66.9–68.3]	0.11^***^	0.10	0.12
35–39	38,558	16.6	49.3[48.5–50.0]	0.05^***^	0.05	0.06
40–44	26,875	11.6	29.5[28.8–30.0]	0.02^***^	0.02	0.03
45–49	18,494	7.9	15.3[14.6–16.0]	0.01^***^	0.01	0.01
**Highest educational level**						
No education	91,834	39.5	69.3[68.7–69.8]	1	-	-
Primary	72,358	31.1	60.0[59.4–60.5]	0.67^***^	0.66	0.68
Secondary	60,054	25.8	66.7[66.1–67.4]	0.90^***^	0.88	0.92
Higher	8538	3.7	65.6[64.1–67.1]	0.81^***^	0.77	0.85
**Partner’s highest educational level**						
No education	79,114	34.0	70.9[70.3–71.5]	1	-	-
Primary	62,759	27.0	59.2[58.6–59.8]	0.59^***^	0.58	0.60
Secondary	73,475	31.6	65.3[64.8–65.9]	0.79^***^	0.77	0.81
Higher	17,436	7.5	66.3[65.3–67.3]	0.79^***^	0.76	0.81
**Parity**						
One birth	39,400	16.9	94.4[94.0–94.7]	1	-	-
Two births	44,071	18.9	83.9[83.3–84.4]	0.33^***^	0.31	0.34
Three births	38,635	16.6	72.4[71.8–73.1]	0.16^***^	0.16	0.17
Four or more births	110,677	47.5	47.1[46.4–47.7]	0.05^***^	0.05	0.06
**Current use of contraceptives**						
No	161,963	69.6	69.3[68.8–69.7]	^1^	-	-
Yes	70,821	30.4	56.8[56.3–57.4]	0.58^***^	0.57	0.59
**Exposure to mass media**						
No	71,829	30.9	68.3[67.8–68.9]	1	-	-
Yes	160,955	69.1	64.2[63.7–64.7]	0.85^***^	0.83	0.86
**Ideal number of children**						
0–3	45,448	19.5	50.8[50.1–51.6]	1	-	-
4–5	85,945	36.9	64.7[64.3–65.2]	0.82^***^	1.78	1.87
6+	101,391	43.6	72.2[71.6–72.7]	2.57^***^	2.51	2.64
**Decision making autonomy**						
Respondent alone	16,056	6.9	48.9[47.7–50.1]	1	-	
Otherwise	216,728	93.1	66.6[66.2–67.0]	1.91^***^	1.85	1.98
**Number of living children**						
0	2321	1.0	96.6[95.6–97.5]	1	-	-
1–3	132,802	57.1	82.2[81.8–82.6]	0.19^***^	0.15	0.23
4+	97,662	41.9	43.3[42.7–43.9]	0.03^***^	0.02	0.04
**Type of place of residence**						
Urban	79,553	34.2	63.4[62.8–64.1]	1	-	-
Rural	153,231	65.8	66.6[66.1–67.0]	1.11^***^	1.09	1.13
**Wealth index**						
Poorest	54,275	23.3	69.1[68.4–69.9]	1	-	-
Poorer	48,520	20.8	66.8[66.1–67.4]	0.90^***^	0.88	0.93
Middle	45,632	19.6	65.1[64.4–65.8]	0.84^***^	0.81	0.86
Richer	43,324	18.6	64.2[63.5–64.9]	0.81^***^	0.79	0.84
Richest	41,033	17.6	62.7[62.0–63.5]	0.77^***^	0.75	0.79

*** = *p* < 0.001; ** = *p* < 0.01 and * =* p *< 0.05, *cOR* crude Odds Ratio, *CI* Confidence Interval

### Statistical analyses

Stata version 14.0 was used to process and analyse the data. The analysis began with a computation of the prevalence of desire for more children in SSA using bar chart. After this, we pooled the datasets and calculated the proportions of desire for more children for each of the explanatory variables. We then used a bivariate logistic regression to assess the association between the explanatory variables and desire for more children. This was done to identify significant explanatory variables for the next part of the analysis, which involved multilevel logistic regression. For the multilevel logistic regression, a two-stage approach was employed, where women were nested within clusters and clusters were considered as random effects to cater for the unexplained variability at the contextual level [31]. Four models were generated from the multilevel modelling, consisting of the empty model (Model 0), Model I, Model II, and Model III. Model 0 showed the variance in desire for more children attributed to the distribution of the primary sampling units (PSUs) in the absence of the explanatory variables. Model I had the individual level factors and desire for more children while Model II contained the contextual level factors and desire for more children. The final model (Model III) was the complete model that had the individual and contextual level factors and desire for more children. Model comparison was done using the log-likelihood ratio (LLR) and Akaike’s Information Criterion (AIC) tests. Odds ratio and associated 95% confidence intervals (CIs) were presented for all the models apart from Model 0. To ensure non-existence of correlation between the significant explanatory variables, we ran a multicollinearity test, using the variance inflation factor (VIF), and the results showed no evidence of collinearity among the explanatory variables (Mean VIF = 1.71, Maximum VIF = 2.93 and Minimum VIF = 1.03**)**. Statistical significance was declared at p < 0.05. Sample weight (v005/1,000,000) was applied to correct for over- and under-sampling while the SVY command was used to account for the complex survey design and generalizability of the findings. According to Hatt and Waters [32], pooling data can reveal broader results that are ‘‘often obscured by the noise of individual data sets.’’ To calculate the pooled values, an additional adjustment is needed to account for the variability in the number of individuals sampled in each country. This is accomplished using the weighting factor 1/(A*n_c_/n_t_), where A is the number of countries asked a particular question, n_c_ is the number of respondents for the country c, and n_t_ is the total number of respondents over all countries asked the question [33].

**Fig. 1:**
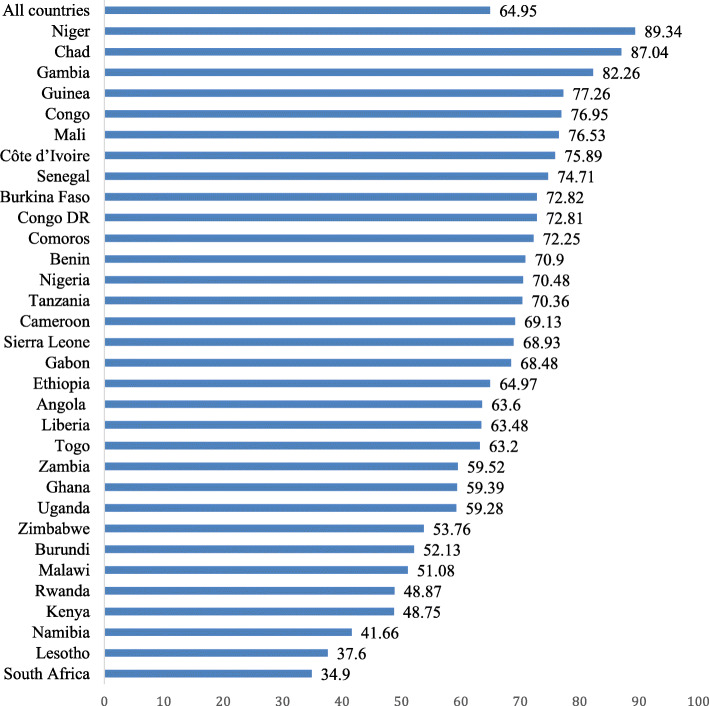
Proportion of women of childbearing women who desire for more children in sub-Saharan Africa

### Ethical approval

The DHSs obtained ethical clearance from the Ethics Committee of ORC Macro Inc. as well as Ethics Boards of partner organisations of the various countries such as the Ministries of Health. During each of the surveys, either written or verbal consent was provided by the women. This was a secondary analysis of data and, therefore, we did not need further approval for this study since the data is available in the public domain. However, we sought permission from MEASURE DHS website and access to the data was provided after our intent for the request was assessed and approved on 3rd April, 2019. Further information about the DHS data usage and ethical standards is available at http://goo.gl/ny8T6X

## Results

The proportion of childbearing women who have desire for more children in SSA is presented in Fig. 1. The overall prevalence of the desire for more children was 64.95%, ranging from 34.9% in South Africa to 89.43% in Niger.

### Bivariate analysis results on desire for more children across socio-demographic characteristics of childbearing women in SSA

The socio-demographic characteristics of the women who participated in the study are presented in Table 2. Table 2 further presents the proportion of women who desire for more children and the unadjusted odds results of the association between the socio-demographic characteristics and desire for more children. Desire for more children was high among women aged 15–19 (95.3%), those with no formal education (69.3%), those whose partners had no formal education (70.9%), women with one birth (94.4%), women who were not current contraceptive users (69.3%), and those who were not exposed to media (68.3%). High prevalence of desire for more children was also found among women who perceived six or more children as the ideal number of children (72.2%), those who do not take decisions alone (66.6%), those who had no living children (96.6%), women who lived in rural areas (66.6%), and poorest women. Results from the bivariate analysis showed that all the independent variables had statistically significant association with desire for more children (see Table 2).

### Multilevel logistic regression results on the predictors of desire for more children among childbearing women in SSA

Table 3 presents results of the multilevel logistic regression analysis of the predictors of desire for children among married and cohabiting women in SSA. With the fixed effects results, results of the individual level predictors showed that women aged 45–49 [AOR = 0.04, CI = 0.03–0.05], those with higher education [AOR = 0.80, CI = 0.74–0.87], and those whose partners had higher education [AOR = 0.88; CI = 0.83–0.94] had lower odds to desire for more children, compared to women aged 15–19, those with no formal education, and those whose partners had no formal education, respectively. Furthermore, the likelihood of desire for more children decreased among women with four or more births [AOR = 0.10, CI = 0.09–0.11], those who were using contraceptives [AOR = 0.68, CI = 0.66–0.70], and those who had four or more living children [AOR = 0.09 CI = 0.07–0.12], compared to those with one child, those who were not using contraceptives, and those who had no living children, respectively. On the other hand, the odds of desire for more children was high among women who considered six or more children as the ideal number of children [AOR = 16.74, CI = 16.06–17.45] and women who did not take decisions alone [AOR = 1.58, CI = 1.51–1.65], compared to those who consider 0–3 children as the ideal number of children and those who took decisions alone, respectively (see Table 3, Model III). With the contextual factors, the odds of desire for more children was high among women who lived in rural areas compared to urban areas [AOR = 1.07, CI = 1.04–1.13].

**Table 3:** Multilevel logistic regression results on the determinants of desire for more children among childbearing women in sub-Saharan Africa

Variables	Null model	Model I	Model II	Model III
**Fixed effects results**				
**Age**				
15–19		1		1
20–24		1.31^***^ (1.18–1.45)		1.31^***^ (1.18–1.45)
25–29		1.19^***^ (1.07–1.31)		1.19^***^ (1.07–1.31)
30–34		0.70^***^ (0.63–0.78)		0.70^***^ (0.63–0.78)
35–39		0.32^***^ (0.29–0.36)		0.32^***^ (0.29–0.36)
40–44		0.12^***^ (0.11–0.13)		0.12^***^ (0.11–0.13)
45–49		0.04^***^ (0.03–0.04)		0.04^***^ (0.03–0.05)
**Highest educational level**				
No education		1		1
Primary		0.72^***^ (0.70–0.75)		0.72^***^ (0.70–0.75)
Secondary		0.78^***^ (0.75–0.81)		0.78^***^ (0.75–0.81)
Higher		0.80^***^ (0.74–0.87)		0.80^***^ (0.74–0.87)
**Partner’s highest educational level**				
No education		1		1
Primary		0.67^***^ (0.65–0.69)		0.67^***^ (0.65–0.70)
Secondary		0.76^***^ (0.74–0.79)		0.77^***^ (0.74–0.80)
Higher		0.88^***^ (0.83–0.93)		0.88^***^ (0.83–0.94)
**Parity**				
One birth		1		
Two births		0.29^***^ (0.28–0.31)		0.29^***^ (0.28–0.31)
Three births		0.12^***^ (0.11–0.13)		0.12^***^ (0.11–0.13)
Four or more births		0.10^***^ (0.09–0.11)		0.10^***^ (0.09–0.11)
**Current use of contraceptives**				
No		1		1
Yes		0.68^***^ (0.66–0.70)		0.68^***^ (0.66–0.70)
**Exposure to mass media**				
No		1		1
Yes		1.01 (0.98–1.03)		1.01 (0.99–1.04)
**Ideal number of children**				
0–3		1		1
4–5		4.35^***^ (4.20–4.51)		4.36^***^ (4.20–4.51)
6+		16.77^***^ (16.09–17.48)		16.76^***^ (16.08–17.48)
**Decision making autonomy**				
Respondent alone		1		1
Otherwise		1.58^***^ (1.51–1.65)		1.58^***^ (1.50–1.65)
**Number of living children**				
0		1		1
1–3		0.26^***^ (0.20–0.34)		0.26^***^ (0.21–0.34)
4+		0.07^***^ (0.06–0.10)		0.07^***^ (0.06–0.10)
**Type of place of residence**				
Urban			1	1
Rural			1.01 (0.99–1.04)	1.07^***^ (1.04–1.10)
**Wealth index**				
Poorest			1	1
Poorer			0.91^***^ (0.88–0.93)	0.97 (0.93-1.00)
Middle			0.84^***^ (0.82–0.87)	0.96 (0.93-1.00)
Richer			0.82^***^ (0.80–0.85)	1.00 (0.96–1.04)
Richest			0.78^***^ (0.76–0.81)	1.03 (0.98–1.08)
**Random effects result**				
PSU variance (95% CI)	0.034 (0.026–0.043)	0.016 (0.011–0.021)	0.03 (0.02–0.04)	0.016 (0.011–0.021)
ICC	0.010	0.005	0.008	0.005
LR Test	χ2 = 283.67***	χ2 = 90.32***	χ2 = 240.51***	χ2 = 91.36***
Wald chi-square	Reference	55040.74***	392.00***	55038.94***
Model fitness				
Log-likelihood	-147612.87	-92583.856	-147416.23	-92570.407
AIC	295229.7	185215.7	294846.5	185198.8
N	232,784	232,784	232,784	232,784

^*^p < 0.05, ^**^p < 0.01, ^***^p < 0.001

*1* Reference category; *PSU *Primary Sampling Unit; *ICC *Intra-Class Correlation; *LR Test *Likelihood ratio Test; *AIC *Akaike’s Information Criterion

Model I adjusted for individual level factors only

Model II adjusted for contextual factors only

Model II adjusted for individual and contextual factors

In terms of the random effects results, in the empty model, there were substantial variations in the likelihood of desire for more children across the clustering of the PSUs (σ2 = 0.03, 95% CI 0.02–0.04). The empty model showed that 3% of the total variance in desire for more children was attributed to between-cluster variation of characteristics (ICC = 0.03). The between-cluster variations showed a decrease from 3–2% from the empty model to the individual-level only model (Model I). From Model I, the ICC increased to 3% (ICC = 0.03) in the contextual level only model but decreased to 2% in the complete model (Model III), which had both the individual and contextual level factors. This explains that the variations in the likelihood of desire for more children could be attributed to the differences in the contextual level factors (see Table 3, Model III).

## Discussion

This study sought to assess the prevalence of desire for more children and its determinants among 232,784 childbearing women in SSA underpinned by the demand-supply framework on fertility by Easterlin [18]. The study found that 64.95% of women desired more children, and this ranged from 34.9% in South Africa to 89.34% in Niger. This finding is comparable to previous studies [34, 35]. However, it is higher than what was found in other studies [36–38]. The possible pathways to explain the differences in the study findings could be differences in study scope and setting, the population sample, and the time these studies were carried out. The higher prevalence of desire for more children recorded in this study could also be attributed to the general importance attached to more children in most parts of SSA [34, 35, 39].

We also observed from this current study that increase in wealth status and educational level of both women and their partners is likely to reduce women’s desire for more children. In addition, women who are working are less likely to desire more children. These findings corroborate several previous studies which have revealed that higher socioeconomic status is associated with lower fertility desires [34, 40–44]. This result can be discussed within the context of the wealth flow hypotheses postulated by Caldwell [45, 46], who elucidates that, in contemporary societies, women and families who are in the high socioeconomic strata tend to view more children as additional burden that has the tendency to strain their resources, including time. On the other hand, women in the low socioeconomic strata might wish to give birth to more children, as they see it as a rational economic decision since they consider each child as an additional asset for security in their old age. Additionally, due to the various economic activities of those in high socioeconomic strata, they might not have more children, as opposed to those who are in the low socioeconomic status who might be into farming activities and consider children as a source of labour for their farming activities.

Channon and Harper [47] also espoused that, in contemporary era, women have competing life goals and this usually translates into less demand for children [17]. They maintained that, for highly educated women, it is sometimes problematic for them to combine many children and life goals such as occupying certain managerial position that will not allow certain amount of maternity leave within a given period. Rabbi [48] also explained that highly educated mothers might be exposed to the various disadvantages associated with high fertility. Similarly, employed mothers always seek for less number of children, as it becomes harder for them to take good care of their children after maintaining the job [48, 49]. The study also showed that women who are in the rural areas are more likely to desire for more children, compared to women in urban areas. This is consistent with what was reported in the context of Bangladesh [48].

Furthermore, the study showed an inverse relationship between age and parity, on the one hand, and desire for more children, on the other hand. Specifically, as parity and age increases, the less likely it is for women to indicate that they desire for more children. This is similar with several previous studies in countries such as China [50] and Sri Lanka [51]. Relatedly, it was found that women who use contraceptives are less likely to desire more children. This corroborates previous studies [39, 52–54]. The probable explanation is that women who are using contraceptives might not wish to give birth to additional children and would adopt various mechanisms to achieve this goal, including the use of contraceptives.

Moreover, it was found that access to mass media is associated with desire for more children. Specifically, women who are exposed to television have lower odds of desiring more children. This finding corroborates previous studies on the association between mass media and fertility behaviours such as small family size [39, 48, 55, 56]. Rabbi [48] explained that messages obtained from television possess a greatest impact on the propensity for people to use family planning methods and also limit the number of children they wish to have, since they can see how beautiful small families are shown on television adverts.

We noted that women having at least six ideal number of children had higher odds of desiring for more children. Having desire for six or more children signifies high demand as espoused by the supply and demand framework [18]. Women with this desire may be of the conviction that they have the reproductive capacity to give birth to the desired higher number “supply”. These women may have a number of rewards for having more children. Among the possibilities to explain this involves the thought that children are assets, gains, or security [57]. Couples with underlying medical conditions such as sickle cells may also be inspired to have more children due to fear of losing some of the children to untimely death [58].

Women without solo decision-making capacity had higher likelihood to desire more children. Being able to decide on matters pertaining to one’s life without any interference offers an opportunity for persons to exercise or implement their choices. Although fertility is declining on the whole [59], the findings of this study indicate that demand is likely to be high if fertility decisions are taken by women alone. The study, therefore, suggests the need for subsequent fertility control interventions to ensure male or partner involvement in fertility decisions. The study also revealed that women with four or more living children were less likely to desire more children. Women with four or more living children may be satisfied with their existing number of children.

### Implications for fertility control

The findings from this study may be instructive to the current fertility control interventions and initiatives across SSA. The study has indicated the traits of women with desire for more children in SSA. Admittedly, nearly all countries in SSA have instituted measures to regulate fertility by moderating both demand and supply factors [60]. Our findings have provided evidence from current data sets that may aid in benchmarking parameters for evaluating and reviewing existing policies and interventions in order to render them more sensitive to the current population. To contextualise our findings and derive much benefit at the country level, governments of SSA and partner organisations should be sensitive to the in-country nuances driven by cultural, geographical, and socioeconomic factors. This may be beneficial in ensuring that the number of children desired by women falls within national estimations and expectations.

### Strengths and limitations of the study

The use of nationally representative datasets of 32 countries in SSA and the multi-stage sampling technique to select the respondents is a major strength of this study. These make it feasible to generalise the findings to all women in sexual unions in SSA. The relatively large sample size also aided in fitting robust logistic regression models to model the factors associated with desire for more children while controlling for confounders. Despite these strengths, it is impossible to establish temporality of sequence, and the possibility of social desirability biases cannot be overruled.

## Conclusion

This study found a relatively high prevalence of women desiring more children. The factors associated with desire for more children are age, educational level, partners’ education, parity, current contraceptive use, ideal number of children, decision-making capacity, number of living children and place of residence. Specific public health interventions on fertility control and those aiming to design and/or strengthen existing fertility programs in SSA ought to critically consider these factors.

## Data Availability

The dataset is available freely for download at: https://dhsprogram.com/data/available-datasets.cfm.

## Abbreviations

cOR: crude Odds Ratio
aOR: Adjusted Odds Ratio
CI: Confidence Interval
SSA: Sub-Saharan Africa
DHS: Demographic and Health Surveys
